# ﻿*Impatienshambaeksanensis* (Balsaminaceae), a new species from South Korea

**DOI:** 10.3897/phytokeys.211.90236

**Published:** 2022-10-21

**Authors:** Ami Oh, Hyun-Do Jang, Jung Sim Lee, Byoung-Un Oh

**Affiliations:** 1 School of Biological Sciences, Chungbuk National University, Cheongju 28644, Republic of Korea Chungbuk National University Cheongju Republic of Korea; 2 Plant Resources Division, National Institute of Biological Resources, Incheon 22689, Republic of Korea National Institute of Biological Resources Incheon Republic of Korea; 3 Division of Forest Biodiversity, Korea National Arboretum, Pocheon 11186, Republic of Korea Korea National Arboretum Pocheon Republic of Korea

**Keywords:** Gangwon-do, Korean endemic, morphology, new species, taxonomy

## Abstract

A new species *Impatienshambaeksanensis* from Gangwon-do, South Korea, is described and illustrated, based on its morphology and distribution. *I.hambaeksanensis* is different from *I.furcillata*, another similar *Impatiens* species in South Korea, in some ways: *I.hambaeksanensis* possesses a serrate leaf margin with flat tooth tip, while *I.furcillata* possesses a crenate leaf margin with erect tooth tip; it has an erect inflorescence, while *I.furcillata* has a pendulous inflorescence; it has a smaller flower which is 2–2.6 cm long, while *I.furcillata* has a flower of 2.3–3.2 cm; the flower is white or pinkish-white with yellowish and purplish spots, while *I.furcillata* has a white lower sepal and pinkish-white lateral united petals with yellowish spots; the distal part of the lower sepal is mostly not coiled or rarely 1-coiled, while that of *I.furcillata* is never coiled; the spur tip is expanded, round and slightly biparted, while that of *I.furcillata* is expanded, ellipsoidal and clearly biparted. A taxonomic description, a holotype and photos of morphological characteristics of the new species are provided. A table which includes the morphological comparison and a geographical distribution map are presented as well.

## ﻿Introduction

Balsaminaceae are a family composed of two genera, which are *Impatiens* and *Hydrocera* (Yuan et al. 2004; Chen et al. 2007). In contrast to *Hydrocera* that is unispecific, *Impatiens* comprises more than 1000 species that are mainly distributed across tropical and subtropical regions (Grey-Wilson 1980; Chen et al. 2007; Xia et al. 2019; Yuan et al. 2022). *Impatiens*, which is known to be taxonomically difficult to study, is distinguished from *Hydrocera* by lateral united petals, valvate fruit and explosive capsule (Grey-Wilson 1980; Chen et al. 2007; Xia et al. 2019).

The first study on Korean *Impatiens* (Balsaminaceae) reported three species, which were *I.textorii* Miq. (Mul-bong-seon in Korean), *I.noli-tangere* L. (No-rang-mul-bong-seon) and *I.furcillata* Hemsl. (San-mul-bong-seon) (Forbes and Hemsley 1886). Subsequently, four to eight *Impatiens* species were recorded in Korea (Nakai 1952; Chung 1956; Lee 1980; Lee 1996; Lee 2006). Most recently, four *Impatiens* species from Korea were reported (Chang et al. 2017). Amongst these recorded species, *I.furcillata* was reported as a new species by Forbes and Hemsley (1886), based on the type materials in the Royal Botanic Garden (K), which were collected in Port Hamilton (officially Geomundo Island in South Korea) and Gensan (Wonsan in South Korea). In previous studies on this species, either simple descriptions, such as, “The overall size is smaller compared to *I.textorii*”, “The spur is long and not coiled” and “The flower is white”, were recorded (Park 1974; Lee 1996) or only the plant list without a description or diagnosis was recorded (Chung et al. 1937; Park 1949; Chung 1956; Lee 1980; Lee 2006). Ji et al. (2010) later re-assessed the taxonomy and morphological characteristics of *I.furcillata*, based on the original description and type materials. They recognised many characteristics that distinguish this species from other Korean *Impatiens* species, including the glabrous stem, drooping peduncle, pinkish–white flower, ovate–oblong leaf blade and non-coiled, biparted spur tip. These characteristics were identical with those of *I.kojeensis* Y.N.Lee (Geo-jae-mul-bong-seon in Korean) and I.hypophyllavar.koreana Nakai (Cheo-jin-mul-bong-seon). Consequently, *I.kojeensis* and I.hypophyllavar.koreana were treated as synonyms of *I.furcillata* and the Korean name of *I.furcillata* was changed to “Cheo-jin-mul-bong-seon” (Ji et al. 2010).

Meanwhile, some morphological characteristics of “San-mul-bong-seon”, have been identified: the overall size is smaller compared to *I.textorii*, the spur is long and not coiled, the peduncle is erect above the leaf and the flower is white (Park 1974; Lee 1996). The present study was conducted to assign a new name to the natural population of “San-mul-bong-seon”, which has been falsely known as ‘*I.furcillata*’, in the Gangwon-do region of the Baekdudaegan Mountain range.

## ﻿Materials and methods

The new species was examined using 20 individual plants (dried vouchers) which were collected in the type locality, the living plants and the immersion specimens in 70% ethyl alcohol collected in the type locality and other habitats (Talbot and White 2013) and the vouchers in the Herbarium of the National Institute of Biological Resources (**KB**) and the Herbarium of the Korean National Arboretum (**KH**) (acronyms after Thiers 2022). In particular, the macro-characteristics, such as the plant height and flower shape, colour and structure, were closely observed and photographed in the habitats. For the morphological observations of micro-characteristics, a light microscope (ECLIPSE E600, Nikon, Japan) and a stereoscopic microscope (LEICA MZ7_5_, Leica, Germany) were used. The immersion specimens for the small structures of the flower and seeds were photographed with scale bar using the stereoscopic microscope and measured, based on microscope magnification.

**Figure 1.**
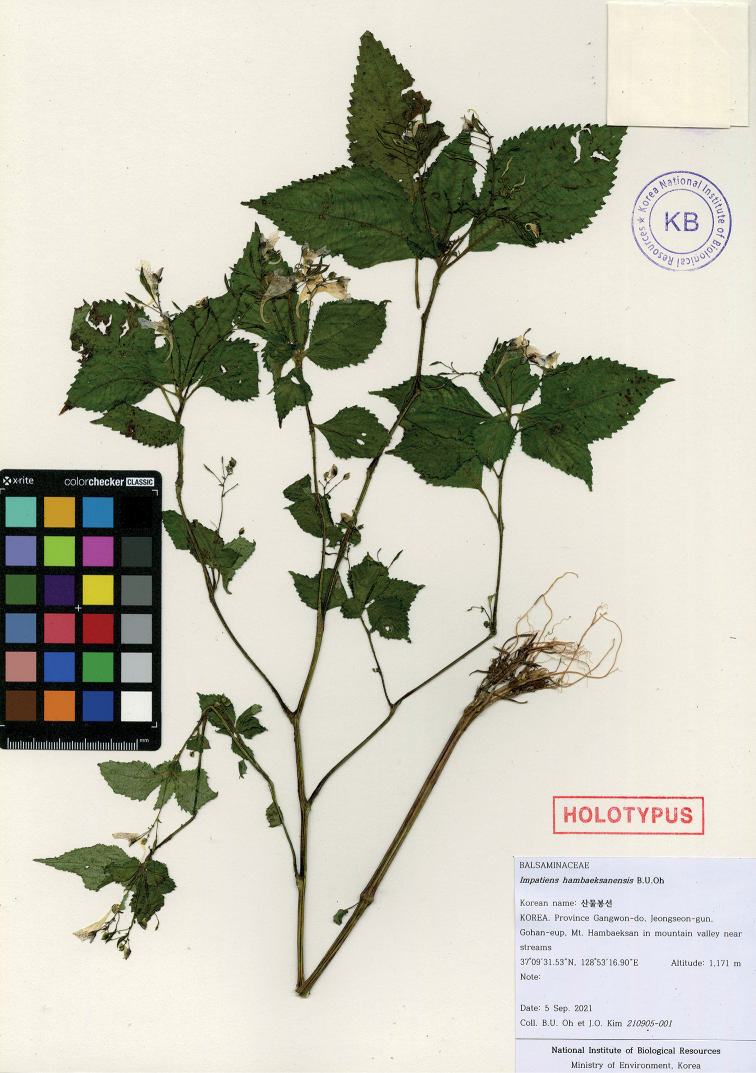
Holotype of *Impatienshambaeksanensis* B.U.Oh, *B.U.Oh & J.O.Kim 210905-001* (KB).

**Figure 2.**
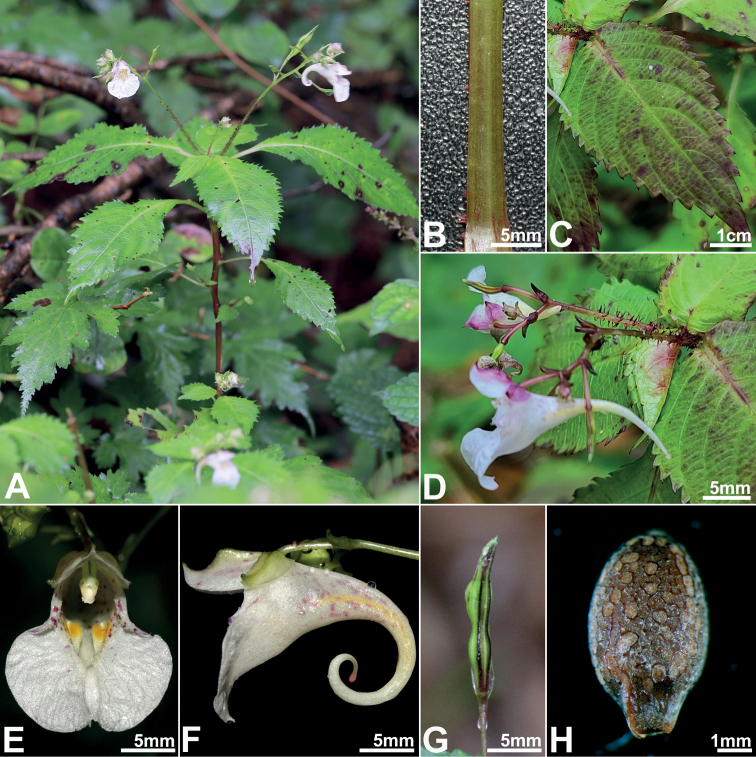
Morphological characteristics of *Impatienshambaeksanensis***A** habit **B** stem **C** leaf **D** inflorescence **E** frontal view of flower **F** lateral view of flower **G** fruit **H** seed. All photos by Byoung-Un Oh.

**Figure 3.**
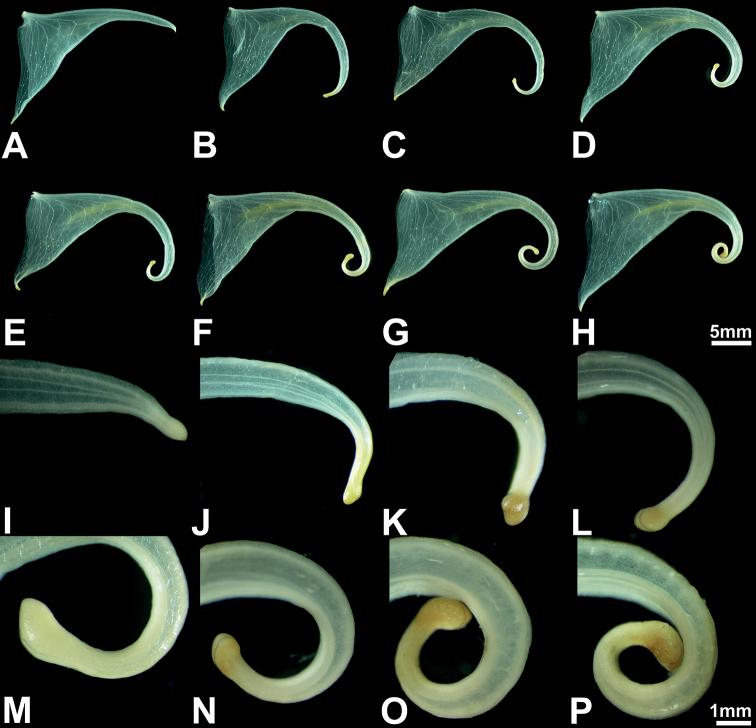
Flower variation ranges of *Impatienshambaeksanensis***A–H** lower sepals **I–P** distal part of lower sepals and spur tips. Scale bars: 5 mm (**A–H**); 1 mm (**I–P**).

**Figure 4.**
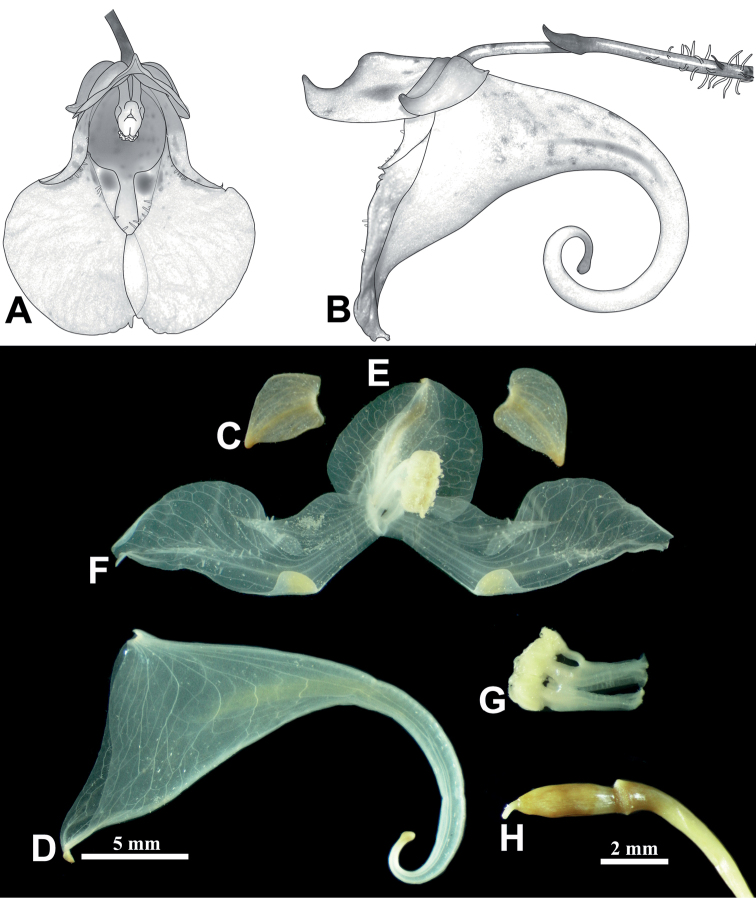
Reproductive organs and their vasculatures of *Impatienshambaeksanensis* (White spots between veins are raphides) **A** frontal view of flower **B** lateral view of flower **C** lateral sepals **D** lower sepal **E** dorsal petal **F** lateral united petals **G** stamen **H** pistil. Scale bars: 5 mm (**D**); 2 mm (**C, E, F, G, H**).

**Figure 5.**
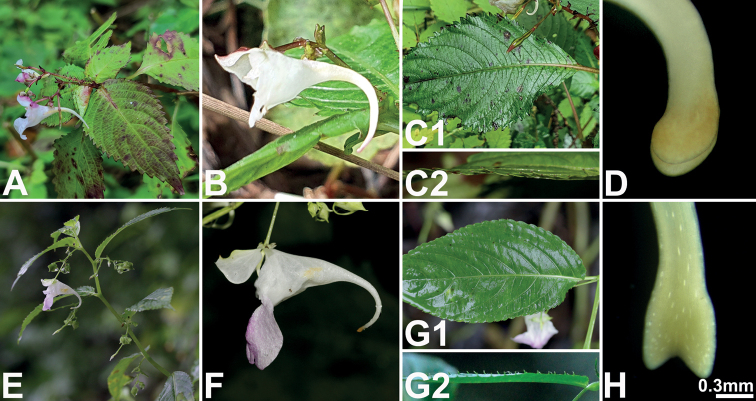
Major morphological differences between *Impatienshambaeksanensis* (**A–D**) and *I.furcillata* (**E–H**) **A, E** inflorescences **B, F** lateral view of flowers and spur tip directions **C, G** leaves (**C1, G1** bird’s-eye view **C2, G2** lateral view) **D, H** shape of spur tips. Scale bar: 0.3 mm (**D, H**). All photos by Byoung-Un Oh.

The morphological description was created using the collected immersion specimens and the vouchers. The figures, which clearly show the taxonomic characteristics of *I.hambaeksanensis*, are provided (Figs 1–4). In addition, we compared the newly-described species with the related taxon, *I.furcillata*, a species that is morphologically most similar to the new species (Table 1; Fig. 5).

**Table 1.** Morphological differences between *I.hambaeksanensis* and *I.furcillata*. Abbreviations. L, Length; W, Width.

Characters	*I.hambaeksanensis*	*I.furcillata*
Leaf shape	elliptic to rhomboid–elliptic	narrowly elliptic to elliptic
Leaf margin shape	serrate	crenate
Leaf tooth tip direction	flat, forward	erect, upward
Inflorescence position	ascending, erect	descending, pendulous
Rachis length (cm)	4–10	0.9–2.2
Rachis trichome type	multicellular multiseriate glandular hair	none
Flower length (cm)	2–2.6	2.3–3.2
Lateral sepal size (L×W, mm)	6 × 4–5	3.5–5.3 × 2.4–4.2
Lateral sepal colour	brownish-white	greenish-white or rarely green
Lower sepal length (mm)	10–18	25–31
Lower sepal colour	white or pinkish-white with yellowish and purplish spots	white with yellowish spots
Lower sepal coiling state	non- to rarely 1-coiled	never coiled
Spur tip shape	round, expanded, slightly biparted	ellipsoidal, expanded, clearly biparted
Dorsal petal size (L×W, mm)	4.8–5.1 × 5.4–6	9–11 × 13–15
Dorsal petal colour	white or brownish-white	greenish-white
Lateral united petal length (mm)	9.5–13	17–24
Lateral united petal colour	white or rarely pinkish-white with yellowish and purplish spots	pinkish-white with yellowish spots
Lateral united petal basal lobe size (L×W, mm)	2.5–4 × 1–2	6.2–7.1 × 4.1–5.1
Lateral united petal distal lobe size (L×W, mm)	7–11 × 3.8–4.4	12–16 × 10–14
Filament length (mm)	ca. 3	3.1–4.5
Anther length (mm)	ca. 1	ca. 2.5
Ovary length (mm)	2.2–2.4	4–4.7
Fruit length (mm)	14–18	15–23

## ﻿Taxonomic treatment

### ﻿ Impatiens hambaeksanensis

Taxon classificationPlantaeEricalesBalsaminaceae

B.U.Oh sp. nov.

EF376DF9-DE4E-5FBD-B3C4-B150318D8996

urn:lsid:ipni.org:names:77306999-1

Figs 1 , 2 , 3 , 4

#### Type.

South Korea. Province Gangwon-do: Jeongseon-gun, Gohan-eup, Mt. Hambaeksan, shady valley near stream in mountainous area, 37°09'31.53"N, 128°53'16.90"E, 1171 m, 5 Sep 2021, *B.U.Oh & J.O.Kim 210905-001* (holotype: KB!; isotypes: KB!, KE!) (Fig. 1).

#### Diagnosis.

*I.hambaeksanensis* is similar to *I.furcillata* in its overall characteristics, including taproot, alternate phyllotaxis and racemose inflorescence, but different from it in some ways: *I.hambaeksanensis* has serrate leaf margin with flat tooth tip; inflorescence is erect; flower is smaller and mostly white or rarely pinkish-white; the distal part of the lower sepal is mostly non-coiled or rarely 1-coiled; the spur tip is expanded, round and slightly biparted.

#### Description.

Herb annual, 42–85 cm tall. Roots taproots. Stems erect, pale green to green or rarely purplish-green, branched, piliferous, with multicellular multiseriate glandular trichomes. Leaves alternate, usually glabrous or having scattered simple trichomes when immature; petioles 2–3.5 cm long; blade green, elliptic or rhomboid–elliptic, 6–11 cm long, 4–6 cm wide, apex acute, base acute or rounded, margin serrate. Bracts triangular, 2.5–4 mm long, 1.5–2 mm wide, glabrous. Inflorescences racemose, axillary; rachises purplish-green, ascending, erect, 4–10 cm long, having dense multicellular multiseriate glandular trichomes; pedicels purplish-white, 0.7–1 cm long, glabrous. Flowers usually white or pinkish-white with yellowish and purplish spots, 2–2.6 cm long, 1.1–1.6 cm wide. Sepals 3; lateral sepals 2, brownish-white, ovate, ca. 6 mm long, 4–5 mm wide; lower sepal 1, white or pinkish-white with yellowish and purplish spots, funnel-form with slender spur, 10–18 mm long, 7–11 mm wide; spur usually not coiled, rarely 1-time coiled, 0.5–0.8 mm long, spur tip expanded, round, slightly biparted. Petals 3; dorsal petal 1, usually white or brownish-white, transversely elliptic, 4.8–5.1 mm long, 5.4–6 mm wide, apex emarginate, base truncate; lateral united petals 2, white or rarely pinkish-white with yellowish and purplish spots, 2-lobed, 9.5–13 mm long; basal lobe white, elliptic, 2.5–4 mm long, 1–2 mm wide; distal lobe white, obovate, 7–11 mm long, 3.8–4.4 mm wide. Stamens 5; filaments linear, upper part connate in a ring around the ovary apex, ca. 3 mm long; anthers white, ovoid, ca. 1 mm long. Pistil 1; ovary fusiform, 2.2–2.4 mm long, glabrous; style very short, ca. 0.5 mm long; stigma 5, beak-like. Fruits capsules, slender, fusiform, 14–18 mm long, glabrous. Seeds 2–5 per capsule, ellipsoidal, brown or dark brown, 4–4.6 mm long, 1.7–2.6 mm wide, surface irregularly reticulate with anticlinal wall. Pollen grains oblong with 4 apertures, 29.4–33.3 µm long, 15.7–21.6 µm wide (Figs 2–4).

#### Distribution and habitat.

**Figure 6.**
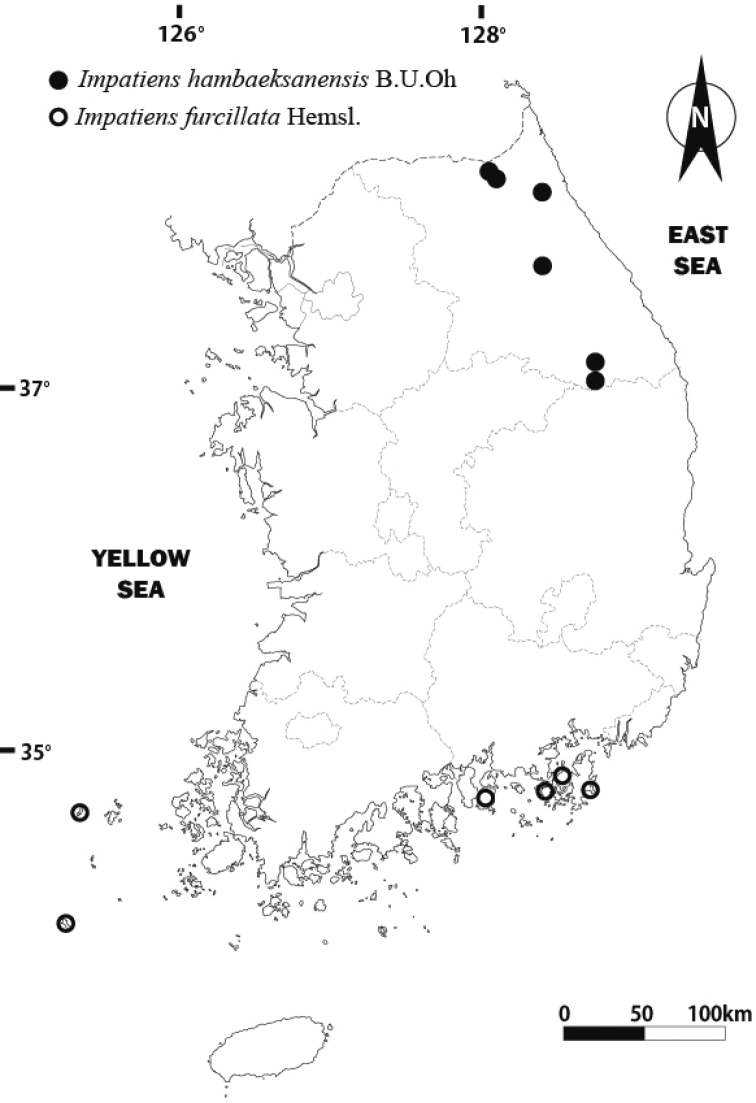
Geographical distribution of *Impatienshambaeksanensis* (●) and *I.furcillata* (○).

In South Korea, *I.hambaeksanensis* is only observed in the central regions, especially in the Baekdudaegan Mountain range, including Gangwon-do (Jeongseon-gun and Yanggu-gun). *I.hambaeksanensis* is generally found in shady valleys or slopes near streams. In contrast, *I.furcillata* is distributed in the southern coastal regions of South Korea (Oh et al. 2016) (Fig. 6). This species is recorded from China and Russia, though it is possible that the plants in those regions were falsely identified as *I.furcillata*, considering their smaller flower which is 0.6–1.8 cm long and the northern limit of *I.furcillata* in South Korea.

#### Phenology.

Flowering was observed from July to October. Fruiting was observed from late July to late October.

#### Conservation status.

Currently, the known habitats of this new species are not legally protected. However, fortunately, many individuals of this species have been detected in the natural populations and the habitats are located in deep mountain valleys. Since the habitats are difficult to access, there may not be problems regarding habitat conservation within the near future. According to the IUCN (2019) Red List Criteria, we suggest that *I.hambaeksanensis* be treated as Data Deficient (DD).

#### Additional specimens examined.

**(*paratypes*)**: South Korea. Gangwon-do: Injae-gun, Mt. Daeamsan, 19 Sep 2021, *LJS21091901* (KH!); Jeongseon-gun, Mt. Hambaeksan, 5 Sep 2012, *KIMJH12157* (KB!), 2 Sep 2015, *Ji S-J et al., sn.* (KH!); Pyeongchang-gun, Mt. Gyebangsan, 23 Aug 2012, *Nam C-H et al. Gyebangsan-120823-035* (KH!); Taebaek-si, Mt. Taebaeksan, 16 Aug 2012, *Byeon J-G et al., sn.* (KH!), 10 Sep 2013, *Yang J-C et al., sn.* (KH!); Yangyang-gun, Mt. Seolaksan, 18 Sep 2018, *KIMJH18092* (KB!); Yanggu-gun, Dolsanryong, 17 Sep 2021, *B.U.Oh & J.O.Kim 210917-001* (KB!).

## ﻿Discussion

For the past 10 years, the authors have attempted to locate the habitats of *I.hambaeksanensis* (San-Mul-Bong-Seon), which has small, white flowers and the characteristic non-coiled spur (Park 1974). Recently, the authors discovered the natural populations, which correspond to *I.hambaeksanensis* in Mt. Hambaeksan and Dolsanryong in the north central region (Gangwon-do) of the Baekdudaegan Mountain range and they confirmed that these were what they had been looking for. In the habitat, most flowers were white and slightly pinkish flowers were seldom observed. In addition, the spur tips were not coiled in most flowers, while in some cases, the tips were coiled once. According to the new classification of the genus *Impatiens*, which was developed by Yu et al. (2016), *I.hambaeksanensis* belongs to the section Impatiens by having racemose inflorescence, 5-carpellate ovary, linear capsule and ellipsoid seed.

According to literature, *Impatienskoreana* Nakai (Nakai 1909) also has white flowers. However, after close examination of the holotype and the isotypes for *I.koreana* Nakai, it was confirmed that this taxon belonged to the natural populations of *I.textorii*. Therefore, *I.koreana* is considered a synonym of *I.textorii*.

In the Korean *Impatiens* species, the expanded spur tip is a taxonomically important characteristic in some cases. For example, *I.hambaeksanensis* has a spur tip that is expanded, round and slightly biparted. Meanwhile, *I.furcillata* has a spur tip that is ellipsoidal, expanded and clearly biparted, with each divided part having a pointed end. The biparted spur tip is considered to have been derived from the round, expanded and unparted tip. In addition, the clearly biparted spur tip of *I.furcillata* appears more evolutionarily advanced than that of *I.hambaeksanensis*.

The flowers of all the *Impatiens* species have coiled spur tips during the early stage of flower development, but as the flower matures, the lower sepal is stretched backwards, thus showing species specificity (Figs 4 and 5). In the case of *I.hambaeksanensis*, the shape of the lower sepal is highly variable and the extent to which the spur tip is coiled varies within a population. However, the spur tip is usually not coiled, except for uncommon cases where it is coiled once (Fig. 3).

Meanwhile, the two syntypes (*Oldham 123*, *Perry 98*) of *I.furcillata* which Hemsley W. previously cited were collected in Port Hamilton (officially Geomundo Island in Korea; *Oldham 123*) and Gensan (Wonsan, an old place name of Jindo Island in Korea; *Perry 98*) in Jeollanam-do (Forbes and Hemsley 1886; Choi 1995). It is also known that *I.furcillata* inhabits restricted southern-coastal regions of low altitude, including Gageodo Island in Shinan-gun, Mt. Cheonkwansan in Jangheung-gun of Jeollanam-do, Mt. Hogusan in Namhae-gun, Mireukdo Island and Mt. Byeokbangsan in Tongyeong-si of Gyeongsangnam-do (Oh et al. 2016). Previously, a plant which is similar to *I.furcillata* was reported in China and Russia. The authors were aware of the existence of this plant in China and Russia and had the opportunity to observe and examine this plant in the field and from the vouchers available. However, this plant has smaller flowers than *I.furcillata*. In addition, from an ecological perspective, it is not probable that *I.furcillata* can exist in China and Russia, considering the northern limit of *I.furcillata* in South Korea, which is the southern coastal region. Therefore, the authors argue that maybe “*I.furcillata*” in China and Russia would have been falsely identified. This species in China and Russia has clearly biparted spur tip which is also observed in *I.furcillata* and this overlapping trait would have led to confusion and false identification.

In contrast, it is known that *I.hambaeksanensis* inhabits mountainous regions of central-northern Korea at elevations of 900–1200 m. Considering these distributional patterns of *I.furcillata* and *I.hambaeksanensis*, it can be concluded that these two species are geographically separated.

## Supplementary Material

XML Treatment for Impatiens hambaeksanensis
